# Spicae aetheroleum in uncomplicated acute bronchitis: a double-blind, randomised clinical trial

**DOI:** 10.1007/s10354-017-0612-0

**Published:** 2017-12-05

**Authors:** Christian Kähler, Tadeusz Derezinski, Joanna Bocian-Sobkowska, Andrea Keckeis, Gabriele Zacke

**Affiliations:** 1 Department for Pneumology, Critical Care and Allergology, Wangen im Allgäu, Germany; 2 Medical Centre ESCULAP, Gniewkowo, Poland; 3 Medical Centre BONUS, Skorzewo, Poland; 4 Medical Centre Bludenz, Bludenz, Austria; 5 Pharmazeutische Fabrik Montavit Ges.m.b.H., Absam, Austria

**Keywords:** Plant preparations, Lavandula, Linalool, Cough, Patient safety

## Abstract

**Background:**

The trial aimed to evaluate the efficacy and safety of Spicae aetheroleum (Spicae ae.), a phytomedicine obtained by steam distillation of the flowering tops of *Lavandula latifolia,* as compared to placebo in adult patients with acute bronchitis.

**Methods:**

Patients with uncomplicated acute bronchitis (bronchitis severity score [BSS] ≥ 5 score points) were randomly assigned to treatment with Spicae ae. or placebo in a double-blind, parallel-group design. No additional treatment was admitted. The primary objective was the mean difference of a defined total BSS of 25% between the Spicae ae. and the placebo group after 7 days of full medication dose. Secondary endpoints included the BSS at day 10, additional signs and symptoms of bronchitis, quality of life (QoL) and safety.

**Results:**

The mean decrease in BSS at day 7 and day 10 was significant with 4.79 vs. 3.20 (*p* < 0.005 for a 25% difference) and 6.47 vs. 4.32 (*p* < 0.009 for a 25% difference) score points respectively in the Spicae ae. (*n* = 119) vs. placebo group (*n* = 110). Accordingly, most additional signs and symptoms of acute bronchitis as well as the patients’ QoL improved significantly with Spicae ae. as compared to placebo. In all, 258 patients were eligible for safety analysis. The treatment with Spicae ae. was well tolerated; no serious adverse events occurred.

**Conclusion:**

The defined objectives both for the primary and secondary endpoints have been met. The results of this study provide evidence that Spicae ae. is well tolerated, effective and superior to placebo in the symptomatic treatment of uncomplicated acute bronchitis in adult patients.

## Introduction

Acute bronchitis is a prevalent, self-limited inflammation of the large bronchi in otherwise healthy subjects that is clinically characterized by cough without pneumonia. Episodes of uncomplicated acute bronchitis are estimated to affect approximately 5% of the general population each year with a higher incidence observed during winter and fall, thus representing one of the most common reasons for medical consultations in primary care and out-patient departments worldwide [1]. Clinical presentation usually includes acute cough (which may or may not be productive) in response to the infection of the bronchial epithelium, wheezing and hoarseness. It may be accompanied by mild fever (≤38 °C) and a general feeling of tiredness. Most of the symptoms commonly resolve within one to three weeks. However, as the associated coughing episodes can persist for up to eight weeks and adversely affect the quality of patients’ lives they constitute one of the most common reasons why patients seek medical advice [2–5].

Due to the lack of a practical test, isolating the causative pathogen is difficult in a routine setting and, indeed, distinguishing a bacterial from a viral infection currently has no bearing on the chosen therapeutic treatment [6, 7]. Nevertheless, up to 90% of episodes of acute respiratory illness in otherwise healthy individuals are considered to be of viral origin [1, 3, 7–9]. Accordingly, the diagnosis of acute bronchitis is based on clinical symptoms and aims to rule out serious conditions (e. g. asthma) and severe bacterial infections, especially pneumonia. In case of a viral infection a causal treatment is impossible. Within this context and the prevailing self-limiting nature of the disease, the treatment of acute bronchitis should focus on symptom relief for the patient by supporting expectoration and thereby alleviating cough. In accordance with treatment guidelines for acute bronchitis a great number of meta-analyses of randomized controlled trials (RCT) and systematic reviews state univocally that a routine prescription of antibiotics to otherwise healthy adults suffering from uncomplicated, acute bronchitis is neither beneficial nor recommended [3, 9–14]. However, studies have shown that antibiotics are prescribed for 60% to 93% of patients diagnosed with this disorder despite potential harms relating to their adverse events and global concerns about the increasing threat of antimicrobial resistance to public health [3, 9, 15]. Thus, healthcare providers/physicians must weigh the benefits and the risk for adverse drug reactions when considering symptomatic therapy. A systematic review of RCTs on the efficacy of non-prescription medications (i. e. antitussives, expectorants, mucolytics and antihistamine-decongestant combinations) compared to placebo revealed conflicting results for the treatment of acute cough in adult patients in community settings, as well as a wide range of mild adverse events [16].

Herbal medicinal products containing plant-derived monoterpenes provide a well-tolerated alternative for the symptomatic treatment of inflammatory airway diseases [17–19]. Several clinical studies showed improved symptom relief in adults suffering from acute bronchitis with plant preparations containing 1,8-cineole and/or linalool when compared to placebo [20–26]. Spike lavender essential oil (Spicae aetheroleum, Spicae ae.) is rich in linalool and 1,8-cineole. Used as a widespread remedy in popular medicine, it is supposed to have antibacterial and antifungal activity, to act secretolytic as well as spasmolytic and to promote expectoration thus inhibiting acute pulmonary inflammation [18, 27–32].

This prospective, multicentre, parallel group, interventional clinical phase IV study aimed to evaluate the efficacy and safety of Spicae ae. as compared to placebo in adult patients suffering from acute bronchitis. Therefore, effects of Spicae ae. on relevant symptoms and on the change of quality of life (QoL) by global assessment scale as well as side effects (incidence and severity) were prospectively evaluated.

## Material and methods

### Study subjects

Patients were eligible to be included in the study if they were ≥18–75 years of age with a Broca Index between 0.75 and 1.30 and evidence of uncomplicated acute bronchitis (≥10 coughing fits during the last day prior to screening visit, Bronchitis Severity Score [BSS] ≥5 points, onset of first symptoms [bronchial mucus production with impaired ability to cough up] within two days before start of treatment, body temperature <39.0 °C; [33]). Patients were excluded with history or presence of confounding respiratory disease (upper respiratory tract infection within the last 4 weeks, chronic bronchitis, chronic obstructive pulmonary disease [COPD], acute exacerbations thereof bronchiectasis, asthma, suspected pneumonia, cystic fibrosis, lung cancer), active cigarette smoking >10/day, concomitant bacterial infection, elevated body temperature (>39.0 °C sublingual), malignant disease of any origin, known or suspected hypersensitivity to the active substance and/or to any of the excipients. Further exclusion criteria included the following: any need for antibiotic treatment in patients at high risk of serious complications because of pre-existing comorbidity, malignancy other than squamous or basal cell carcinoma of the skin; antibiotic therapy (local or systemic) at any time during the preceding four weeks; need for administration of concomitant local and systemic medications including antibiotics, corticosteroids and antihistaminic agents; immunosuppressive therapy; radiation therapy or chemotherapy within the previous 12 months; pregnancy or breastfeeding; history of alcohol or drug abuse likely to lead to uncooperative behaviour; history of psychiatric and/or neurological illness likely to lead to uncooperative behaviour; participation in a clinical research study within the last 6 weeks; evidence or suspicion of non-compliance; inability to provide informed consent and patients using medication for treatment of common-cold-like symptoms (excluding nasal douche). Apart from saline inhalation no other concomitant medications were allowed for relief of bronchitis symptoms. Participants were recruited by general practitioners, specialists of pneumology or by hospital doctors from pneumology clinics from eight study centres located in Austria (3 centres) and Poland (5 centres).

The study was approved by the responsible Ethics Committees and registered at the European Medicines Agency EudraCT number: 2013-004836-31. All patients provided written informed consent. The trial was conducted according the Declaration of Helsinki, the Good Clinical Practice guidelines of the International Conference on Harmonization, and relevant local national laws and regulations concerning clinical trials. All local legal requirements regarding data protection were adhered to.

### Study design

The primary objective was the mean difference of a defined total BSS of 25% between the verum group (active substance: Spicae ae.) and the placebo group after 7 days (±1 day in case of prevention) of full medication dose. Secondary objectives evaluated the mean difference of a defined total BSS of 25% between the verum group and the placebo group after 10 days of full medication dose; the global impact of disease on QoL after 7 and 10 days of full medication dosage and the incidence and severity of adverse events and toxicities as reported by the patient or observed by the investigator. Additionally, the progression of the most common accessory symptoms of acute bronchitis (i. e. impairment of general condition, difficulty swallowing, hoarseness, headache, pain in limbs and joints, fatigue, sore throat and acute rhinitis) was assessed in both groups after 7 and 10 days of full medication dosage.

The sample size calculation was based on information from previous trials and taking into account the objectives of this trial. According to the most conservative value observed in a trial published by Matthys and Heger we assumed a standard deviation of 3.2 for the mean change of BSS score from day 0 to day 7 [34]. It was established that a sample size of *n* = 130 (including a drop-out rate of 20% plus a buffer of *n* = 21) in each group allows the detection of a minimum difference of 25% in BSS after 7 days of treatment with a power of 90% using the Mann–Whitney test (2-sided, alpha-level 0.05). Statistical programming and analysis were performed using SPSS® version 23.0 (IBM, SPSS, Armonk, NY, USA).

Data were recorded with the aid of case report forms (CRF). One CRF had been filled in by the physician for each patient. Data required in the CRF had to be recorded at the beginning of therapy (day 0), at the second visit after 7 days of treatment and the third visit after 10 days of therapy (end of therapy, EOT).

### Methods

Pharmazeutische Fabrik Montavit Ges.m.b.H., Austria, conducted the study and provided study medication. Verum capsules (batch no. 13449501) with gastroresistant coating contained 150 mg Spicae aetheroleum (Tavipec®, manufacturer: Pharmazeutische Fabrik Montavit Ges.m.b.H., Absam, Austria) as the active ingredient; placebo capsules (batch no. 11536401) with gastroresistant coating were filled with medium-chain triglycerides (manufacturer: Catalent®, Eberbach, Germany). Spicae ae. is a herbal medicinal product containing the essential oil obtained by steam distillation of the flowering tops and stalks of *Lavandula latifolia* as the active ingredient. Its main constituents are linalool, 1,8-cineole and camphor in concentrations of 34–50%, 16–39% and 8–16%, respectively, as sourced from the European Pharmacopoeia 8^th^ edition (http://online6.edqm.eu/ep800/). Spicae ae. is authorised to the market in Austria since 1959 but not yet authorised in Poland.

The administration route for all study medications was oral application. Study participants were assigned to swallow 2 capsules three times daily every day as a whole with some liquid, 30 min before a meal (breakfast, lunch, dinner) for 10 days. The dose schedule was planned according to the summary of product characteristics (SmPC) recommendations and the same for all patients. The investigational medicinal products (IMPs) were supplied in a double blind way according to a randomization list prepared by computer (random permuted blocks, confidential block size) via Rancode Professional® (IDV Gauting, Germany). Each patient was provided capsules for 10 days (+2 days in case of weekend). Both, verum and placebo were indistinguishable with regard to capsule size and outer appearance, smell, packaging and labelling. Adherence was tracked by counting the number of remaining capsules from each individual patient, both by the study nurse and the study monitor (four eye principle). As a characteristic ethereal taste may be perceptible during therapy with Spicae ae., Austrian participants to the study had to be naïve to Spicae ae. in order to avoid unblinding by the patient.

### Analysis

Data required in the CRF had to be recorded at the beginning of therapy, at the second visit after 7 days of treatment and the third visit after 10 days of therapy (end of therapy, EOT). Efficacy was recorded using the BSS, derived by summing responses to five major symptoms (i. e. cough, sputum, rales/rhonchi, chest pain during coughing, dyspnoea) with higher scores indicating more severe symptomatology, rated from 0 to 4 (0 = absent, 1 = mild, 2=moderate, 3 = severe, 4 = very severe). Further signs and symptoms of acute bronchitis (i. e. impairment of general condition, difficulty swallowing, hoarseness, headache, pain in limbs and joints, fatigue, sore throat and acute rhinitis) were assessed by verbal rating from 0 to 3 with higher scores indicating more severe symptomatology (0 = none, 1 = slight, 2 = moderate, 3 = severe). The global impact of disease on QoL was verbally assessed by the question “How troublesome are your symptoms of bronchitis?” At any visit, patients should assess their condition on a 10-point scoring system ranging from “Not troublesome” (= 0) to “Worst thinkable troublesome” (= 10). Scores from 0–3, 4–7, and 8–10 indicated mild, moderate and severe impact, respectively. Adverse events were either reported by the patient or observed by the investigator (recorded at each visit). As far as possible, each adverse event was described by its duration (start and end dates), its severity (mild, moderate, severe), its relationship to the study medication (not related, unlikely, possibly, probably, definitely), whether this influenced the course of the study medication (continued per protocol, reduced, interrupted, discontinued, therapy change), or whether it required specific therapy. No interim analysis was planned or performed.

All patients who completed the defined course of treatment for the primary (7 days) and secondary endpoints (10 days) were eligible for the efficacy analysis. All subjects included into the study, who had received at least one dose of medication and who provided at least one post-baseline safety information were considered evaluable for safety analysis (Intent to treat [ITT] principle). For efficacy parameters, all analyses were done for the per-protocol (PP) population of the secondary endpoint (10 days). The main efficacy variable was quantitative, however, not necessarily normally distributed; therefore a two-sided (α = 5%) Mann–Whitney test (rank-sum test) was applied. The differences in scores for the secondary endpoints were analysed with the Mann–Whitney test analogously. All secondary parameters were summarized using descriptive statistics, i. e. number (%) of patients for categorical variables and mean, SD (standard deviation), median, minimum/maximum for continuous variables. Descriptive statistics were produced by treatment group.

## Results

**Fig. 1:**
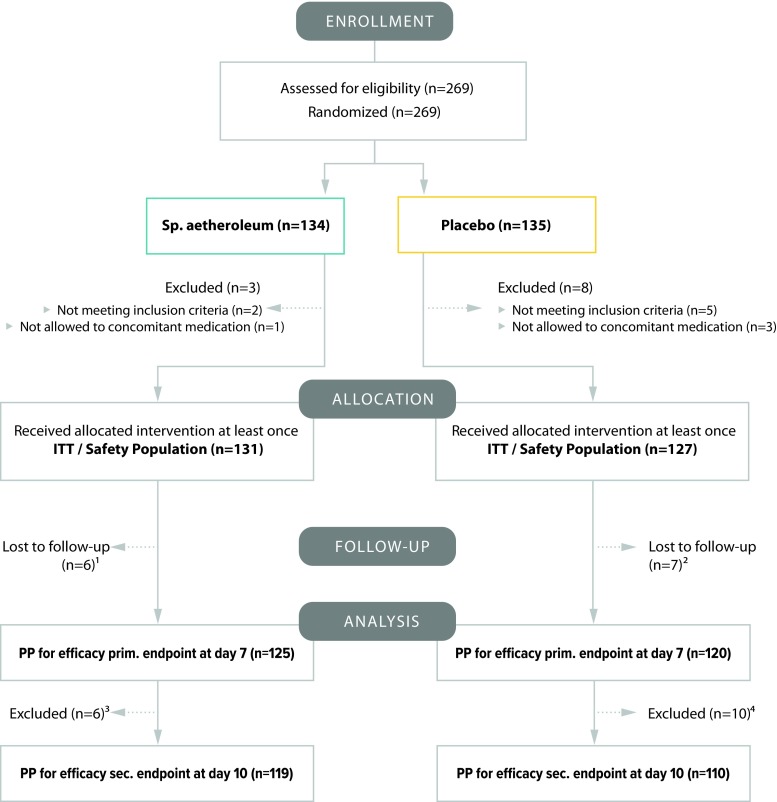
Flow diagram. ^1^ 6 patients stopped treatment before visit; 2 due to failure, 1 patient withdrawal of IC, 2 patients no visit, 1 patient due to AE. ^2^ 1 patient hospitalization (severe AE, not related to study medication), 2 patients stop of treatment before visit 2 due to failure; 3 patients not compliant; 1 patient withdrawal of IC. ^3^ 6 patients stopped treatment on/after visit 2 due to failure and need for AB treatment. ^4^ 6 patients stopped treatment on/after visit 2 due to failure and need for AB treatment; 2 patients withdrawal of IC; 2 patients not compliant. *AB* antibiotic; *AE* adverse event; *ITT* intention to treat; *PP* per protocol, *IC* informed consent, *prim.* primary, *sec.* secondary

From May 2014 to January 2016 a total of 269 patients were enrolled and randomly assigned, 11 patients did not start treatment. In total, 258 patients fulfilled the inclusion criteria and received at least one application of study medication thus constituting the ITT population and the safety analysis subset (Fig. 1). Thirteen patients were lost to follow-up and were excluded from the ITT population; hence 245 patients completed the defined course of treatment for the primary endpoint (7 days). The per-protocol subset equal to the subset for the efficacy analysis was formed by 229 patients who completed the whole treatment schedule (10 days).

The baseline characteristics were balanced within both groups (Table 1). Of the 258 patients 49.2% (*n* = 127) were female and 50.8% (*n* = 131) were male. The onset of first symptoms of bronchitis occurred at least one day before study entry and start of treatment. Apart from saline inhalation no other concomitant medications, herbal and complementary medicine use were allowed for symptom relief.

**Table 1:** Baseline demographic characteristics of patients (Intent-to-Treat Population)

Characteristic	Spicae aetheroleum (SD)	Placebo (SD)	All (SD)
Patients, *n* (M:F)	131 (72:59)	127 (59:68)	258 (131:127)
Age, years	40.4 (13.1)	40.4 (15.0)	40.4 (14.1)
Body weight, kg	75.5 (13.5)	73.8 (14.0)	74.7 (13.7)
Body height, cm	173.1 (10.1)	172.1 (10.0)	172.6 (9.8)
Broca Index	1036 (0.1)	1024 (0.1)	1030 (0.1)
Body temperature, °C	37.4 (0.6)	37.4 (0.6)	37.4 (0.6)
BSS	8.38 (0.2)	8.18 (0.2)	8.28 (0.1)

Data are presented as mean (range), exception: patients (ratio, male:female). Broca Index = actual weight/height in cm − 100; *BSS* Bronchitis Severity Score, *F* female, *M* male, *SD* standard deviation

### Efficacy analysis

The defined objectives both for the primary and secondary endpoints have been met.

**Fig. 2:**
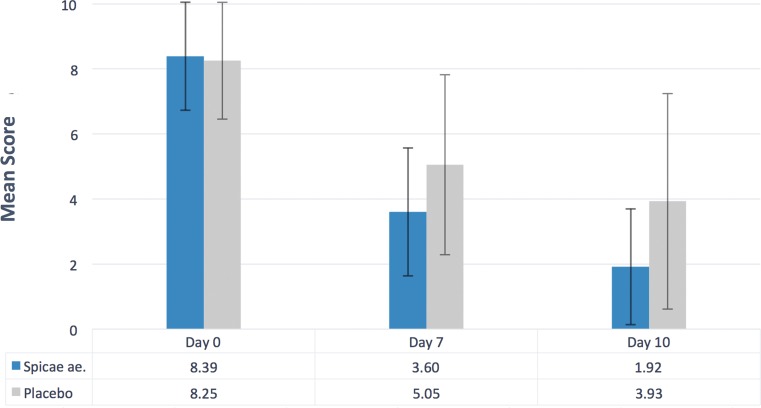
Mean BSS at day 0, day 7 and day 10. Mean BSS at different treatment days (*n* = 229; Spicae ae. *n* = 119, placebo *n* = 110). The mean decrease in BSS at day 7 was significant (*p* < 0.005 for a 25% difference). The mean decrease at day 10 was significant (*p* < 0.009 for a 25% difference). *BSS* Bronchitis Severity Score

The evaluation of the primary efficacy parameter (*n* = 229) revealed a significant improvement of the individual signs and symptoms of bronchitis as reflected in the BSS at day 7 after full medication dose with Spicae ae. in comparison to treatment with placebo (*p* < 0.005 for a 25% difference; Fig. 2). During a 7-day treatment course the BSS improved by a mean of 4.79 (57.1%) and 3.20 score points (38.8%) in the Spicae ae. and the placebo group, resulting in a mean difference of 1.6 (95% confidence interval [CI] 1.01–2.19) score points, respectively.

The analysis of the secondary efficacy endpoint at day 10 (*n* = 229) revealed a significantly lowered BSS in the Spicae ae. group compared to the placebo group (*p* < 0.009 for a 25% difference). The mean difference in improvement of the BSS after a 10-day treatment between the Spicae ae. (77.1%) and the placebo group (52.4%) accounted for 2.2 (95% CI: 1.48–2.82) score points (6.47 vs. 4.32, respectively).

**Fig. 3:**
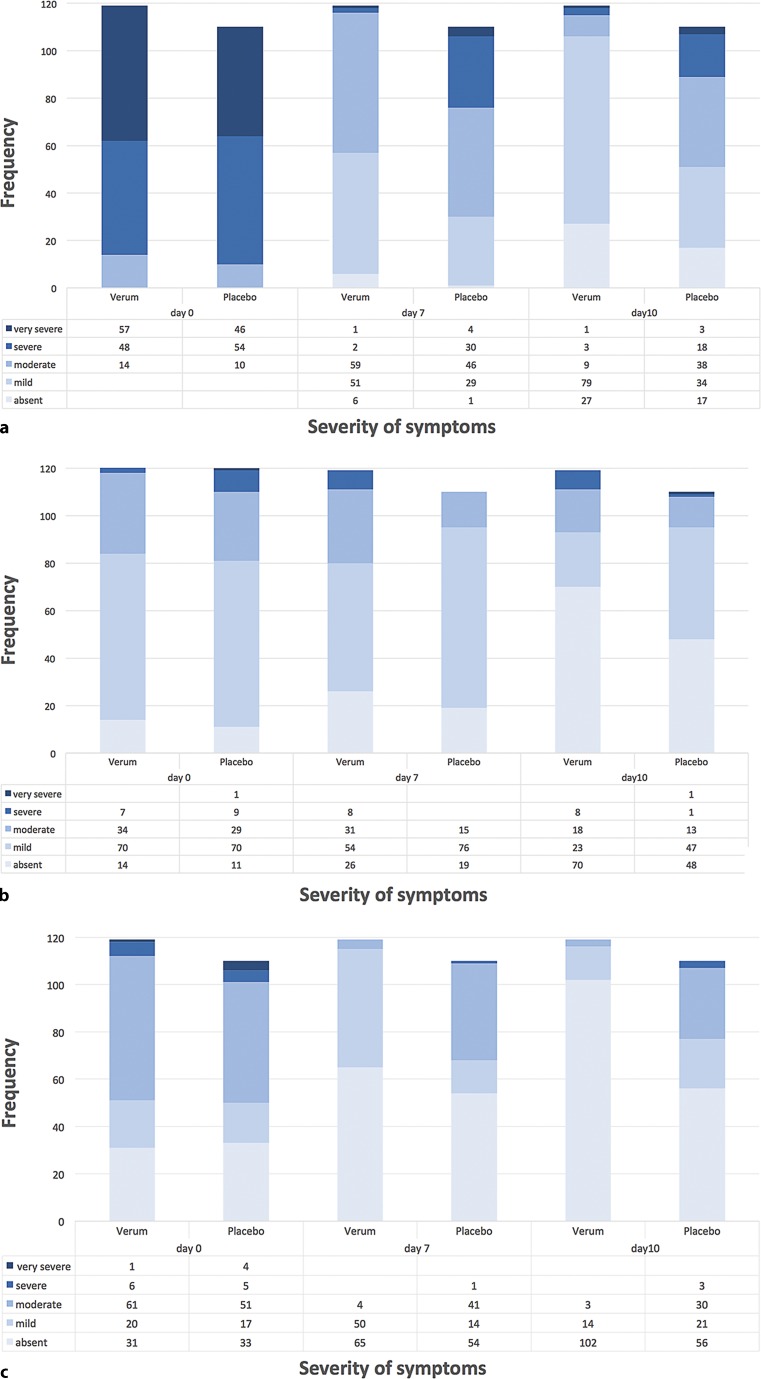
Categorized outcome of individual BSS parameters at day 0, day 7 and day 10. Patient frequencies for the respective category (absent, mild, moderate, severe, very severe) of signs and symptoms of bronchitis composing the BSS at different treatment days (*n* = 229; Spicae ae. *n* = 119, placebo *n* = 110). χ^2^ tests of independence for the frequency for category and the treatment groups revealed significance: **a** Cough (day 7: *p* < 0.000001; day 10: *p* < 0.000000001) **b** Sputum (day 7: *p* < 0.001, day 10: *p* = 0.001) **c** Rales/Rhonchi (day 7: *p* < 0.0000000001; day 10: *p* < 0.0001). *BSS* Bronchitis Severity Score

**Fig. 4:**
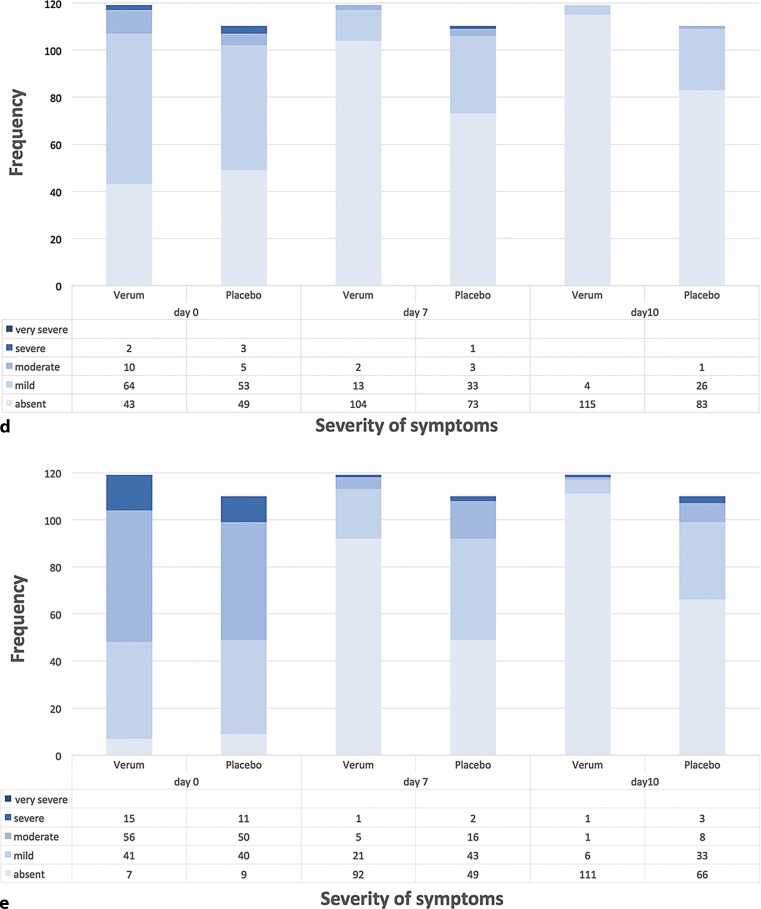
Categorized outcome of individual BSS parameters at day 0, day 7 and day 10. Patient frequencies for the respective category (absent, mild, moderate, severe, very severe) of signs and symptoms of bronchitis composing the BSS at different treatment days (*n* = 229; Spicae ae. *n* = 119, placebo *n* = 110). χ^2^ tests of independence for the frequency for category and the treatment groups revealed significance: **d** Dyspnoea (day 7: *p* = 0.002; day 10: *p* = 0.00002). **e** Chest pain during coughing (day 7: *p* < 0.00001; day 10: *p* < 0.0000001). *BSS* Bronchitis Severity Score

The categorized outcomes of the individual symptoms forming the BSS (i. e. cough, sputum production, rales/rhonchi, chest pain during coughing and dyspnoea) measured at day 0, day 7 and day 10 are depicted in Fig. 3 and 4.

All relevant parameters for the BSS were ameliorated during the treatment with Spicae ae. as compared to placebo. Except for sputum production the improvement of all remaining parameters reached significance with Spicae ae., both at day 7 and day 10 of study treatment. At baseline cough was the predominant symptom in both groups, being severe or very severe in 88.2% of the Spicae ae. and 90.9% of the placebo patients. At day 7, the proportion of patients with severe or very severe cough had dropped to 2.5% in the Spicae ae. group versus 30.9% in the placebo group. Similarly, at baseline 94.1% of patients in the Spicae ae. group and 91.8% of patients in the placebo group suffered from chest pain during coughing. During therapy with Spicae ae. at day 7 chest pain subsided in 77.3 vs. 44.5% of patients in the placebo group, respectively. At EOT, 93.3 vs. 60% of the Spicae ae. vs. placebo patients were free of chest pain. Rales on auscultation affected 73.9% in the Spicae ae. and 70.0% in the placebo group at baseline. After 10 days of treatment with Spicae ae. 85.7% vs. 50.9% of placebo patients got rid of rales/rhonchi. During therapy the proportion of patients with symptoms of dyspnoea decreased by 51.3 vs. 21.9% at day 7 and by 60.5 vs. 31.0% at EOT in the Spicae ae. and placebo group as to baseline, respectively. Sputum production was comparable between both groups, affecting around 90% of patients at first visit (day 0) and 41.2 vs. 56.4% of patients in the Spicae ae. and placebo group, respectively, after 10 days of treatment.

In accordance with the improvement of the BSS after 7 and 10 days of treatment with Spicae ae. the evaluation of the most common accessory symptoms of acute bronchitis also showed a statistically significant amelioration. All investigated parameters (i. e. impairment of general condition, difficulty swallowing, hoarseness, headache, pain in limbs and joints, fatigue, sore throat and acute rhinitis) as assessed by verbal rating by the patient showed a significant difference between the Spicae ae. group and the placebo group both after 7 days (acute rhinitis *p* = 0.005; residual parameters: *p* < 0.0001) and after 10 days (all: *p* < 0.0001). After 7 days of treatment the general condition was still impaired in 85.5% of the placebo patients compared to 68.1% of Spicae ae.-treated patients. About half of the patients (49.1%) who received the placebo presented with difficulty in swallowing at this time, in contrast to only 17.6% of the Spicae ae.-treated patients. At the end of therapy still 31.8% of the placebo patients suffered from slight difficulty swallowing compared to only 4.2% in the Spicae ae. group.

### Assessment of quality of life

**Fig. 5:**
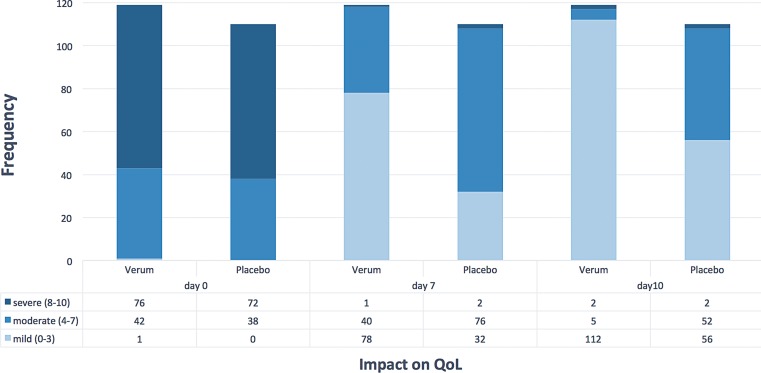
Quality of life after 7 and 10 days of treatment. Patient frequencies for quality of life (QoL) at different treatment days (*n* = 229; Spicae ae. *n* = 119, placebo *n* = 110) verbally assessed by the question “How troublesome are your symptoms of bronchitis?”, rated by a 10-point scoring system ranging from “not troublesome” (= 0) to “worst thinkable troublesome” (= 10). Scores from 0–3, 4–7 and 8–10 indicating mild, moderate and severe impact on QoL, respectively. The χ^2^ tests of independence for the changes in categories and the treatment groups from day 0 to day 7 or day 10 was significant: *p* = 0.00002 at day 7 and *p* = 0.000006 at day 10

Fig. 5 depicts the number of patients which changed the respective category (mild = 0–3, moderate = 4–7 and severe = 8–10) during the time course of treatment. The difference in changes between categories from baseline to day 7 (*p* = 0.00002) or day 10 (*p* = 0.000006) was significant between Spicae ae. and placebo. The improvement across two categories, i. e. from severe to mild impact on QoL, occurred significantly more frequent with Spicae ae. vs. placebo both after 7 and 10 days of treatment (*p* < 0.00001). The individual assessment of QoL by global assessment scale revealed that the impact of the disease on mean QoL both, at day 7 and day 10 was significantly lower after treatment with Spicae ae. as compared to the placebo (*p* < 0.0001 and *p* < 0.0001, respectively; Fig. 5). At baseline about two thirds of all patients (*n* = 229) were severely affected (mean QoL score = 7.67). At the end of therapy, 94.1% of the evaluable patients who were treated with Spicae ae. reported to be mildly impacted in contrast to 50.9% in the placebo group.

### Safety evaluations

From 269 randomized patients, 258 (131 males, 127 females) received at least one dose of the allocated intervention (including placebo) and were eligible for safety analysis (ITT population). More than 80% of patients in the Spicae ae. (82.4%) and the placebo group (86.6%) were exposed to study medication for ≥10 days, ingesting a mean (median) of 56 (58) capsules. The treatment with Spicae ae. was generally well tolerated. Thirteen patients in the Spicae ae. and nine patients in the placebo group reported in total 27 adverse events (AEs), of which 17 were regarded to be possibly related (Table 2). Twelve of 131 patients (9.2%) in the Spicae ae. group vs. 5 of 127 patients (3.9%) in the placebo group showed possibly related drug-related adverse events (AEs) including gastrointestinal disorders, skin and subcutaneous tissue disorders, respiratory, thoracic and mediastinal disorders. In three patients treatment was discontinued prematurely because of the occurrence of an AE. However, no AE was defined as definitely related and there were no probably related AEs. One serious unrelated AE (1/127, 0.78%) was reported in the placebo group.

**Table 2:** Reported adverse events by severity

	Spicae ae. (*N* = 131)			Placebo (*N* = 127)		
	Mild	Moderate	Severe	Mild	Moderate	Severe
**Surgical and medical procedures**						
Hospitalization	–	–	–	–	–	1 (0.8)
**Gastrointestinal disorders**						
Abdominal discomfort	–	–	–	1 (0.8)	–	–
Abdominal pain	6 (4.6)	1 (0.8)	–	5 (3.9)	–	–
Nausea	3 (2.3)	–	–	1 (0.8)	–	–
Vomiting	–	–	–	–	1 (0.8)	–
**Musculoskeletal & connective tissue disorders**						
Back pain	–	–	–	1 (0.8)	–	–
Back thoracic pain	–	–	–	–	1 (0.8)	–
**Infections and infestations**						
Oral herpes	1 (0.8)	–	–	–	–	–
**Injury, poisoning & procedural complications**						
Trauma of left foot	1 (0.8)	–	–	–	–	–
**Skin and subcutaneous tissue disorders**						
Rash	2 (1.5)	–	–	–	–	–
**Respiratory, thoracic & mediastinal disorders**						
Rhinitis allergic	1 (0.8)	–	–	–	–	–
**Ear and labyrinth disorders**						
Hypoacusis	–	–	–	–	1 (0.8)	–
Total	14 (10.7)	1 (0.8)	–	8 (6.3)	3 (2.4)	1 (0.8)

Data are presented as *n* of adverse events with % of patients reporting in parentheses

## Discussion

This study represents the first randomized, placebo-controlled phase IV study to determine the efficacy and safety of Spicae ae. capsules in adult patients suffering from acute bronchitis using the BSS, composed from the individual scores for cough, sputum, rales/rhonchi, chest pain during coughing and dyspnoea. When compared to placebo, the patients in the Spicae ae. group had a distinct benefit both in terms of amelioration of the most important symptoms comprising the BSS and in terms of concomitant complaints in the course of acute bronchitis. The early alleviation of symptoms and the observed significant improvement of most accessory symptoms of acute bronchitis in the Spicae ae. group became further apparent in terms of the significantly ameliorated QoL score as verbally assessed by the patients. The dosage regimen of two capsules thrice a day was well tolerated and no increase in serious complications was observed compared to placebo.

The results of this study provide evidence that Spicae ae. is effective and superior to placebo in the symptomatic treatment of acute bronchitis in adult patients. The BSS was explicitly designed to assess the clinical status of a patient with acute bronchitis at various points of time, i. e. baseline and follow-up visits based on physician-assessed items in conjunction with subjective feedback from the patient [35]. The steady improvement of symptoms over time in the placebo group is a well-known phenomenon corroborating the self-limiting nature of the disease: In all the placebo-controlled studies on effects of medications, the BSS total score under placebo decreased significantly from the first to the final visit [35]. However, most patients suffering from acute bronchitis still seek medical advice due to persisting cough and substantial symptomatic discomfort [2, 4, 8, 36]. Investigations on the appropriate treatment of cough revealed that the patients’ pressure and expectations lead a majority of physicians to the unnecessary prescription of antibiotics [8, 37] although several guidelines and meta-analyses have found no benefit in the use of antibiotics for the treatment of acute bronchitis [3, 5, 8, 9, 14, 38, 39]. A recent Cochrane review meta-analysis including 17 clinical trials exposed no difference in clinical improvement when antimicrobial treatment was compared with placebo [3].

In order to provide rapid symptom relief for patients suffering from acute bronchitis, herbal remedies and their monoterpenoid constituents (e. g. cineole, linalool, camphor) may constitute a well-tolerated and well-evidenced alternative to the prescription of antibiotics, thus not only reducing antibiotic use but also the global risk of bacterial resistance development. The results of our investigation correspond with outcomes from other controlled trials that investigated comparable herbal preparations [20–26] containing 1,8-cineole and other monoterpenes as active substances. In a double-blind, randomized, placebo-controlled trial on acute bronchitis Gillissen et al. found consistently better efficacy for Myrtol standardized (Myrtol s., the main component being cineole) than for placebo with respect to the mean change in coughing fits from baseline to about one week treatment (*p* < 0.0001). The median time to 50% reduction in coughing fits was significantly shorter and there were significantly less day-time coughing fits, less difficulty coughing up, and less sleep disturbance due to night-time coughing when compared to placebo [21]. The observations made by Gillissen et al. conform to our findings: After 7 and 10 days of treatment with Spicae ae. the patients reported a significant amelioration of cough as well as significantly reduced chest pain during coughing compared to those in the placebo group. Matthys et al. randomly allocated 676 patients suffering from acute bronchitis to a 2-week treatment course with either Myrtol s., cefuroxime, ambroxol or placebo in a double-blind, parallel-group fashion. The responder rates after one week of treatment were statistically significantly higher (*p* < 0.001) for Myrtol s. as compared to placebo and similar to those for cefuroxime and ambroxol. Moreover, for several ancillary criteria Myrtol s. tended to be superior to cefuroxime and ambroxol [23]. It is interesting to note that the decrease in sputum production with Spicae ae. did not reach significance. After holding steady from baseline to day 7 it trended to improve at day 10 similar to placebo. The delayed sputum reduction might be an indication for the secretolytic action of Spicae ae. [40, 41]. A cough producing significant quantities of sputum should usually not be suppressed in order to maintain the distribution of alveolar ventilation and the protective barrier of the bronchi. Against the background of the significant improvement of all other measured concomitant symptoms and complaints, especially of acute cough and chest pain during coughing, the apparent sputum reduction after 7 to 10 days of treatment may be related to the anti-inflammatory properties of the main ingredients of Spicae ae.: linalool and 1,8-cineole [18, 19, 31, 42, 43].

Apart from Spicae ae., linalool is also a major component in thyme. A study conducted by Kemmerich et al. treated 361 patients suffering from acute bronchitis and revealed superiority (*p* < 0.0001) with a thyme herb and ivy leaves combination compared to placebo with regard to reduction of the frequency of coughing fits [26]. The regression of symptoms was faster and the responder rates compared to placebo were significantly higher when treated with the thyme–ivy combination. The authors concluded that the positive results obtained with the herbal preparation correspond well with the pharmacological actions, which are secretolytic, expectorant, broncho-spasmolytic, antibacterial and antiphlogistic in the case of thyme/linalool. Ancient investigations inferred that essential oils containing cineole as well as cineole as monosubstance may improve mucociliary clearance and clinical symptoms. Indeed, 1,8-cineole, the second main component of Spicae ae., as well as other monoterpenes, were effective for symptomatic treatment of acute rhinosinusitis and bronchitis as well as bronchial asthma in randomized clinical trials [21, 23, 41, 44, 45]. The two main components of Spicae ae., 1,8-cineole and linalool, are believed to mediate its anti-inflammatory, expectorant and spasmolytic actions as well as the antiseptic, antihemolytic and antimicrobial effects; however, some of these actions have been investigated only preclinically [18, 27–32, 42, 43, 46, 47]. Hence, the efficacy of Spicae ae. in other respiratory disorders may be deduced and further investigation in this field is required. Currently, a multicentre RCT is investigating the efficacy and safety of Spicae ae. in the treatment of acute rhinosinusitis.

We are aware that the characteristic ethereal taste, which is perceptible during therapy with Spicae ae., may have affected the blinding in our study. Therefore Austrian participants had to be naïve to the active substance in order to be eligible for randomization. However, the lack or presence of the perception of an ethereal taste may have influenced some study participants thus representing a limitation of our study. In order to provide a representative cohort of patients that has its main source in primary care and to facilitate earliest possible therapeutic intervention neither chest radiograph nor microbiological testing of the sputum was mandatory in the diagnosis of acute bronchitis to participate in this RCT. Clinical guidelines on appropriate antibiotic use recommend to refrain from further testing unless pneumonia is suspected [9]. Accordingly, in our study the initial diagnosis of acute bronchitis primarily aimed to rule out pneumonia. Furthermore, additional clinical data (e. g. forced expiratory volume, FEV_1_) were collected voluntarily by numerous medical centres (data on file). The validation of the used parameter BSS is based on several studies on acute bronchitis [21, 22, 25, 33, 34]. The baseline BSS in our study was comparable to the severity of illness as reflected in the baseline BSS from other interventional studies [22, 25, 26, 34]. Therefore, we assume that the patients who participated in our study constitute a representative sample of the patient collective suffering from acute bronchitis; thus the external validity of our study can be deduced.

The adverse events observed in this study were mostly of mild severity and in accordance with the known safety profile of Spicae ae. as described in the literature. Fifteen of the possibly treatment-related adverse events involved the gastrointestinal tract, the skin and the respiratory system. One serious adverse event was noticed in the placebo group; however, it was not considered to be related to treatment. No specific areas of concern or safety signals have been identified in the included patients. Adverse events such as hypersensitivity reactions and gastrointestinal tract reactions are known adverse drug reactions of Spicae ae. capsules, listed in the SmPC and were counterbalanced in this study by the significant amelioration of signs and symptoms of acute bronchitis as compared to placebo. With the use of antibiotics the frequency and severity of gastric disorders, skin reactions and possible anaphylactic reactions is clearly higher: 5–25% develop antibiotic-associated diarrhoea, 2% develop skin reactions, 1 of 5000 will have an anaphylactic reaction [13].

The results of this study indicate that Spicae ae. effectively improves the signs and symptoms of uncomplicated acute bronchitis in adult patients. The intake of two capsules thrice daily is feasible and well-tolerated. In accordance with the current treatment guidelines the present study contributes additional evidence suggesting that Spicae ae. constitutes an appropriate therapeutic option to reduce the prescription of antibiotics for acute bronchitis apart from the strict indication for an antibiotic therapy.
